# Serum free and bioavailable rather than total testosterone is associated with the progression of non-alcoholic fatty liver disease in postmenopausal women: a prospective study

**DOI:** 10.3389/fendo.2026.1741229

**Published:** 2026-04-29

**Authors:** Xu Wang, Qing Li, Jiesheng Lin, Zhijun Pan, Yupeng Zeng, Tianran Shen, Xu Chen, Wenhua Ling, Yuming Chen

**Affiliations:** 1 Department of Clinical Nutrition, Guangdong Provincial People’s Hospital (Guangdong Academy of Medical Sciences), Southern Medical University, Guangzhou, Guangdong, China; 2 Guangdong Provincial Key Laboratory of Food, Nutrition and Health, Guangzhou, Guangdong, China; 3 School of Public Health, Sun Yat-sen University, Guangzhou, Guangdong, China; 4 School of Public Health and Emergency Management, School of Medicine, Southern University of Science and Technology, Shenzhen, Guangdong, China; 5 Department of Nutrition and Food Hygiene, School of Public Health, Guangdong Pharmaceutical University, Guangzhou, Guangdong, China; 6 Guangdong Engineering Technology Center of Nutrition Transformation, Guangzhou, Guangdong, China

**Keywords:** androgen, bioavailable testosterone, cohort study [or longitudinal study], non-alcoholic fatty liver disease (NAFLD), free testosterone (FT), postmenopausal women, sex hormone

## Abstract

**Background:**

Sex steroid hormones may play vital roles in predisposing individuals to metabolic diseases. However, relationship between testosterone and progression of non-alcoholic fatty liver disease (NAFLD) in postmenopausal women remains unclear. Therefore, we conducted a community-based prospective cohort study, to investigate the associations between NAFLD progression and serum testosterone, as well as its fractions, in postmenopausal women.

**Methods:**

A total of 1,705 postmenopausal women were included and analyzed after 5,269 person-years of follow-up. Serum total testosterone (TT) was measured by chemiluminescent immunoassay; concentrations and percentages (%) of free testosterone (cFT) and bioavailable testosterone (BioT) were calculated. Their relationships with NAFLD development or regression were investigated using logistic regression.

**Results:**

Baseline concentrations and percentages of cFT and BioT, rather than TT levels, were significantly higher in non-NAFLD postmenopausal women who developed NAFLD after follow-up than in those who did not, while prominently lower in women with NAFLD regression than in those who had sustained NAFLD. Moreover, both higher concentrations and percentages of cFT and BioT were associated with the increased risk of NAFLD development; the fully adjusted ORs (95% CIs) for Q4 *vs*. Q1 of cFT were 2.35 (1.44–3.84) and 4.58 (2.67–7.86), and for BioT were 1.95 (1.21–3.14) and 3.13 (1.87–5.24). Meanwhile, they were associated with the decreased probability of NAFLD regression, the fully adjusted ORs (95% CIs) of cFT were 0.56 (0.33–0.95) and 0.23 (0.12–0.42), of BioT were 0.54 (0.31–0.91) and 0.22 (0.12–0.40). However, no significant association between TT levels and NAFLD progression was observed.

**Conclusions:**

Serum concentrations and percentages of cFT and BioT are associated with the progression of NAFLD among postmenopausal women; they could be potential biomarkers for predicting NAFLD progression. Further studies in diverse populations and with a longer follow-up period are needed to verify whether our findings have broader implications.

## Introduction

Non-alcoholic fatty liver disease (NAFLD), which is now also known as metabolic associated fatty liver disease (MAFLD) or metabolic dysfunction-associated steatotic liver disease (MASLD), has become one of the most common chronic liver diseases worldwide, affecting up to 32% of the overall adult populations at the moment (1, 2). The wide spectrum of NAFLD extends from simple steatosis through non-alcoholic steatohepatitis (NASH) to advanced fibrosis, cirrhosis, and ultimately hepatocellular carcinoma (3). To make things worse, a staggering proportion of NAFLD patients have one or more metabolic comorbidities, which can further increase the risk of type 2 diabetes, cardiovascular diseases and extrahepatic cancers considerably (4–6).

The pathogenesis of NAFLD is complex and multifactorial (7, 8). Recently, it has been proposed that NAFLD is regarded as a sexual dimorphic disease, as researchers have already found the sex-specific differences in NAFLD epidemiology (9, 10). In addition, evidence from NAFLD patients and zebrafish with experimental steatosis showed that ovarian senescence facilitates both the development of massive hepatic steatosis and the fibrotic progression of liver disease (11). It is suggested that postmenopausal women, when compared with premenopausal women and men of the same age, are more prone to metabolic imbalances, and may lose the protection of estrogen against developing severe hepatic steatosis and fibrosing NASH (12). Therefore, given the dramatic rise in NAFLD prevalence and liver-related mortality due to NASH cirrhosis (13), there is a growing need to identify the key risk factors and the novel noninvasive biomarkers for early warning of NAFLD progression in postmenopausal women.

There is no doubt that endocrine derangements are closely linked with dysmetabolic traits. Accumulating evidence suggests that sex steroid hormones play vital roles in predisposing individuals to cardiovascular and metabolic diseases (14–16). Meanwhile, females are usually more sensitive to fluctuations in reproductive hormone levels (17). As we know, testosterone is secreted mainly by the male testicles, and to a lesser extent, by the female ovaries and placental tissues. It exerts physiological effects on both reproductive and nonreproductive tissues in females. Therefore, for women, testosterone is also an essential hormone, with its physiological actions mediated directly or via aromatization to estradiol throughout the body (18).

Some observational studies have already explored the relationships between circulating TT and NAFLD, and the results from male patients were relatively consistent. Nevertheless, data in the literature concerning these relationships in females are inconsistent (19–23). Different study populations and diagnostic methods, cohorts based on specific diseases, and limited sample sizes are all possible factors for these conflicting results. In the meantime, cross-sectional or case-control studies cannot address the cause-effect relationship between testosterone and NAFLD progression. Notably, testosterone circulates in plasma in three different forms: unbound (free), albumin-bound, and sex hormone-binding globulin (SHBG)-bound. However, most of the existing research only focused on the total level. To shed light on this issue, we conducted this community-based prospective study in postmenopausal women to investigate the associations between NAFLD progression and serum TT level, together with serum concentrations and percentages of calculated free testosterone (cFT) and bioavailable testosterone (BioT). Clarifying these questions can help us to find out whether circulating testosterone can predict the development or regression of NAFLD and be of great importance for a better management of NAFLD in postmenopausal women.

## Participants and methods

### Study design, participants recruitment, and follow-up

**Figure 1:**
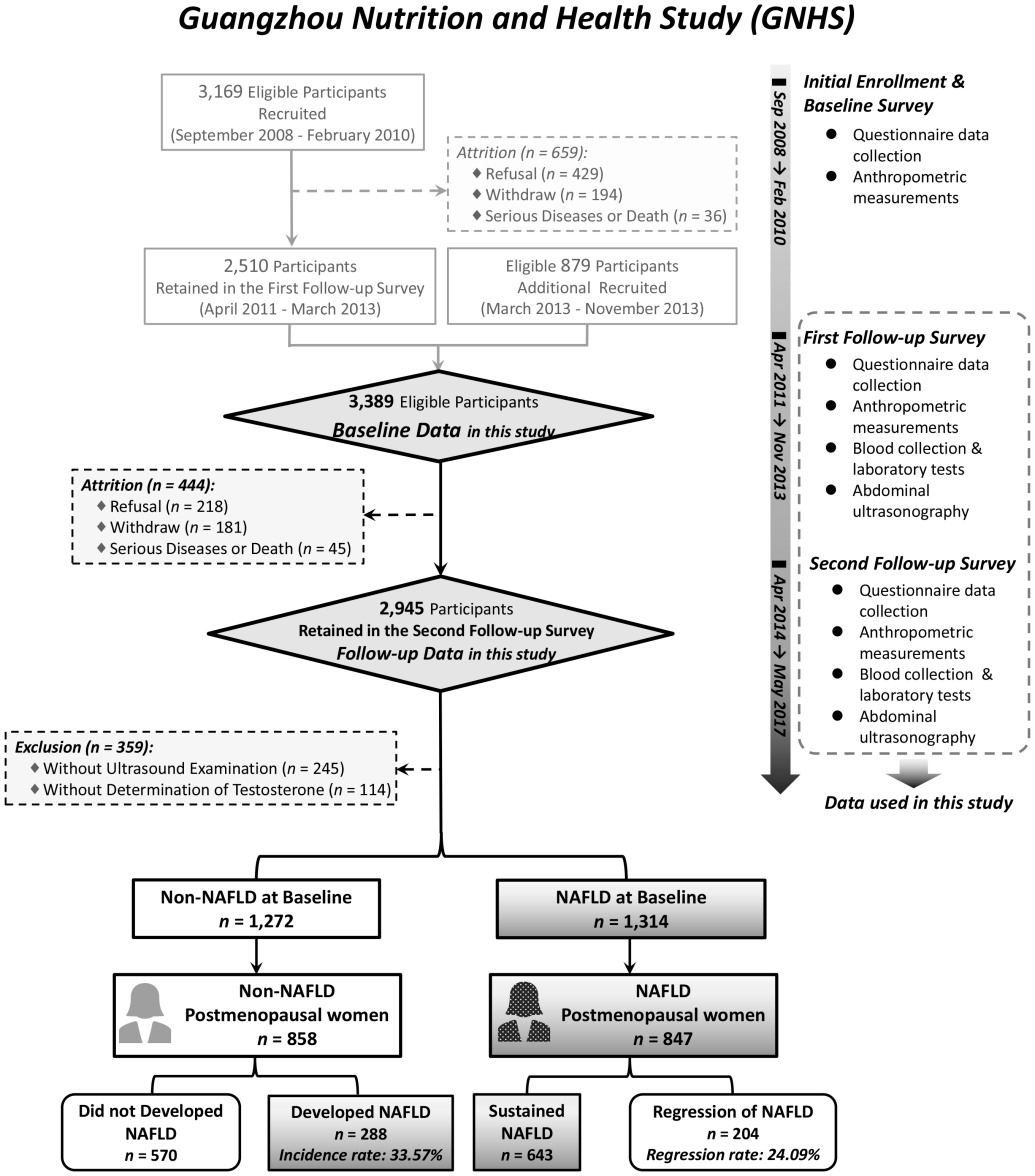
Flow chart and timeline of the recruitment and the follow-up process of participants. A total of 3,169 eligible participants were initially recruited, and 2,510 of them retained in the first follow-up survey. An additional 879 participants were recruited to account for participant attrition. In this cohort study, the Baseline Data were derived from the surveys of these two groups of participants. After a mean duration of 3.09 ± 0.41 years, 2,945 participants finished the second follow-up survey. The Follow-up Data in this study were derived from the second follow-up survey, while the timeline listed the items that we collected in each survey. At last, a total of 847 non-alcoholic fatty liver disease (NAFLD) postmenopausal women and 858 non-NAFLD postmenopausal women were enrolled in the final analysis.

Our study was implemented based on the Guangzhou Nutrition and Health Study (GNHS), a community-based prospective cohort study in China (ClinicalTrials.gov, NCT03179657). The study was approved by the Ethics Committee of the School of Public Health at Sun Yat-sen University (ZDGWYL2009-3) and conducted in accordance with the Declaration of Helsinki. Before the initial enrolment, all participants underwent a comprehensive face-to-face questionnaire survey and anthropometric assessment, which have been described previously (24). The exclusion criteria were as follows: excessive alcohol consumption (≥ 140 g/week for males or ≥ 70 g/week for females), drug- or toxin-induced liver diseases, autoimmune or any viral hepatitis, genetic liver diseases, biliary obstructive diseases, any cancers, HIV or hepatitis virus infection, chronic kidney diseases, current treatment with systemic corticosteroids or anti-inflammatory drugs, or pregnancy. Postmenopausal status was defined as self-reported natural amenorrhea for at least 12 consecutive months prior to enrollment, consistent with the WHO and STRAW + 10 criteria (25). To validate the reliability of self-reported menopausal status, we cross-referenced participants’ reported status with age. The flow chart of the recruitment and follow-up process is presented in **Figure 1**. All eligible participants signed the written informed consent and were instructed to maintain their lifestyle and dietary patterns.

### Biological sample collection and laboratory measurements

Venous blood samples were collected between 8:00 and 10:00 am from participants after overnight fasting, on the same day as the ultrasonography examination. Serum biochemical indices, including fasting glucose, triglycerides (TG), total cholesterol (TC), high-density lipoprotein cholesterol (HDL-C), low-density lipoprotein cholesterol (LDL-C), aspartate aminotransferase (AST), alanine transaminase (ALT), alkaline phosphatase (ALP), albumin, uric acid (UA) and high-sensitivity C-reactive protein (hsCRP), were measured using a Hitachi 7600–010 automated analyzer (Hitachi, Tokyo, Japan). Serum fasting insulin was measured by microparticle enzyme immunoassay using kits obtained from Abbott Corporation (Chicago, IL, USA). The homeostasis model assessment (HOMA) of insulin resistance (IR) was calculated as fasting glucose (mmol/L) × fasting insulin (*μ*U/ml)/22.5.

### Determination of serum total, free, and bioavailable testosterone levels

Chemiluminescent microparticle immunometric assay using kits obtained from Abbott Corporation were used to measure serum TT, SHBG and dehydroepiandrosterone sulphate (DHEAS) levels with an Abbott ARCHITECT i2000 automated analyzer (Chicago, USA). The lower limits of detection for TT, SHBG and DHEAS were 4.33 ng/dL, 4.5 nmol/L and 4.8 *μ*g/dL, respectively. The intra-assay coefficients of variation for TT, SHBG and DHEAS were 2.0–5.1%, 4.1–7.7% and 1.4–4.2%, respectively. Levels of cFT and BioT were calculated using the Vermeulen equations (26). In addition, the free androgen index (FAI) was calculated as TT (nmol/L)/SHBG (nmol/L) × 100, and the percentages of cFT and BioT in TT were calculated as %cFT = cFT (ng/dL)/TT (ng/dL) × 100%, and %BioT = BioT (ng/dL)/TT (ng/dL) × 100%.

### Clinical diagnosis of NAFLD

Abdominal ultrasonography was performed using a Sonoscape SSI-5500 Doppler sonography (Shenzhen, China). Scanning was conducted by the same group of experienced sonographers using a standardized protocol who were blinded to the participants’ clinical data. The working definition of NAFLD in this study was according to the criteria issued by the Chinese Liver Disease Association: 1) liver imaging findings that meet the diagnostic criteria for diffuse fatty liver and have no other causes to explain; and/or 2) patients with components related to metabolic syndrome who exhibit unexplained persistent elevation of serum ALT and/or AST, γ glutamyl transpeptidase for more than half a year (27). Moreover, we determined the inter-operator reliability for the ultrasound NAFLD evaluations in 100 participants, and the results showed good precision (Spearman’s *r* = 0.911, *kappa* = 0.875, and total agreement = 93%, *P* < 0.001). In the meantime, validity was assessed in another 34 participants who underwent further evaluations by abdominal computed tomography, and the image diagnosticians were blinded to the ultrasound results. We also observed good agreement between the validity assessment samples (Spearman’s *r* = 0.905, *kappa* = 0.691, and total agreement = 85%, *P* < 0.001).

### Statistical analyses

Data management and statistical analyses were performed using SPSS 25.0 (IBM Inc., Chicago, USA). Since the first follow-up, we began to perform the abdominal ultrasonography. Therefore, in the present study, our Baseline Data were derived from the first follow-up survey, while the Follow-up Data were derived from the second follow-up survey (**Figure 1**). Normally distributed data were expressed as means ± SD and compared using Student’s *t*-tests, while non-normally distributed data were reported as medians [25th, 75th percentiles] and compared using Mann-Whitney *U*-tests. Proportions were compared using Chi-square tests. Furthermore, multivariate logistic regression models were used to separately evaluate associations between three different fractions of testosterone and the progression (development or regression) of NAFLD, with adjustment for confounding variables. Potential confounders were selected based on strong biologic justification and were determined according to both univariate results and literature reports. Testosterone levels were treated as either categorical variables (by quartiles) or continuous variables after standardization (presented per 1-SD increase). The subgroup analyses by age (< 60 *vs*. ≥ 60 years), body mass index (BMI, < 24.0 *vs*. ≥ 24.0 kg/m^2^), homeostasis model assessment of insulin resistance (HOMA-IR, < 2.0 *vs*. ≥ 2.0), and triglycerides (TG, < 1.7 *vs*. ≥ 1.7 mmol/L) were further performed to test whether the results were consistent across different subgroups. Furthermore, multiple stepwise logistic regression analyses were used to identify the independent predictors of NAFLD progression. A two-tailed *P-value* < 0.05 was considered statistically significant.

## Results

Except for 359 participants who did not undergo ultrasound examination at the follow-up survey (*n* = 245) or whose baseline testosterone and SHBG levels were not measured (*n* = 114), 1,272 non-NAFLD participants (384 men and 888 women) and 1,314 NAFLD participants (436 men and 878 women) completed all examinations after a 3-year follow-up (**Figure 1**). Of the postmenopausal women at baseline, 858 did not have NAFLD, while 847 were diagnosed with NAFLD; together, they were enrolled in the final analysis of this prospective study.

### Clinical parameters and testosterone levels of postmenopausal women without NAFLD at baseline

As shown in **Table 1**, postmenopausal women who developed NAFLD after follow-up had higher BMI, waist-to-hip ratio (WHR), trunk fat mass, fasting insulin and glucose, HOMA-IR, TG, LDL-C to HDL-C ratio, UA, DHEAS, and a higher prevalence of hypertension and dyslipidemia (all *P-value* < 0.01), but had lower HDL-C, SHBG, and AST to ALT ratio (all *P-value* < 0.05) than those who were not diagnosed with NAFLD after follow-up.

**Table 1:** Basal clinical parameters, serum testosterone levels of postmenopausal women distributed by the progression status (development or regression) of NAFLD at follow-up.

Variables	Overall (n = 1,705)	Non-NAFLD at baseline			NAFLD at baseline		
		Sustained non-NAFLD at follow-up (n = 570)	Developed NAFLD at follow-up (n = 288)	P-value	Regressed to non-NAFLD at follow-up (n = 204)	Sustained NAFLD at follow-up (n = 643)	P-value
Demography							
Age (year)	59.95 ± 5.13	59.57 ± 5.03	59.65 ± 5.11	0.825	60.48 ± 5.58	60.26 ± 5.07	0.615
Menopausal duration (year)	10.30 ± 5.95	9.99 ± 5.50	10.19 ± 6.01	0.117	11.05 ± 6.90	10.39 ± 5.97	**0.044**
Household income (CNY/month/person)				0.625			0.503
≤ 3000	1,250 (73.31%)	419 (73.51%)	212 (73.61%)		160 (78.43%)	459 (71.38%)	
3001–6000	347 (20.35%)	120 (21.05%)	58 (20.14%)		31 (15.20%)	138 (21.46%)	
> 6000	80 (4.69%)	21 (3.68%)	16 (5.56%)		7 (3.43%)	36 (5.60%)	
Unknown	28 (1.64%)	10 (1.75%)	2 (0.69%)		6 (2.94%)	10 (1.56%)	
Anthropometry							
BMI (kg/m2)	23.35 ± 3.13	21.38 ± 2.49	23.12 ± 2.33	**< 0.001**	23.61 ± 2.62	25.10 ± 3.06	**< 0.001**
WHR	0.91 ± 0.07	0.89 ± 0.07	0.93 ± 0.07	**< 0.001**	0.90 ± 0.07	0.94 ± 0.06	**< 0.001**
Trunk fat mass (kg)	10.01 ± 2.76	8.15 ± 2.31	9.85 ± 2.08	**< 0.001**	10.38 ± 2.32	11.62 ± 2.47	**< 0.001**
SBP (mmHg)	123.07 ± 18.09	118.95 ± 17.76	120.97 ± 16.93	0.105	125.11 ± 17.53	127.02 ± 18.15	0.179
DBP (mmHg)	74.13 ± 10.21	71.73 ± 10.37	72.80 ± 9.30	0.128	74.92 ± 9.88	76.61 ± 9.99	**0.035**
Blood biochemical indexes							
Fasting insulin (μU/ml)*	7.84 [5.55, 11.39]	5.78 [4.18, 7.74]	7.42 [5.71, 10.38]	**< 0.001**	7.84 [5.76, 10.22]	10.65 [7.88, 15.18]	**< 0.001**
Fasting glucose (mmol/L)*	4.75 [4.34, 5.25]	4.68 [4.27, 5.11]	4.76 [4.39, 5.25]	**0.003**	4.62 [4.20, 5.13]	4.90 [4.49, 5.45]	**< 0.001**
HOMA-IR*	1.68 [1.13, 2.51]	1.21 [0.86, 1.68]	1.67 [1.20, 2.26]	**< 0.001**	1.64 [1.15, 2.16]	2.38 [1.63, 3.42]	**< 0.001**
TG (mmol/L)*	1.25 [0.91, 1.74]	1.01 [0.76, 1.37]	1.25 [0.90, 1.76]	**< 0.001**	1.13 [0.88, 1.59]	1.58 [1.15, 2.16]	**< 0.001**
TC (mmol/L)	5.73 ± 1.05	5.74 ± 0.99	5.76 ± 1.15	0.803	5.68 ± 1.01	5.72 ± 1.06	0.622
LDL-C (mmol/L)	3.68 ± 0.91	3.64 ± 0.85	3.67 ± 1.04	0.777	3.74 ± 0.89	3.71 ± 0.91	0.735
HDL-C (mmol/L)	1.51 ± 0.41	1.68 ± 0.44	1.50 ± 0.36	**< 0.001**	1.54 ± 0.39	1.34 ± 0.34	**< 0.001**
LDL-C/HDL-C	2.62 ± 0.93	2.32 ± 0.86	2.58 ± 0.96	**< 0.001**	2.56 ± 0.86	2.91 ± 0.90	**< 0.001**
AST (U/L)*	19.00 [16.00, 22.00]	19.00 [17.00, 23.00]	18.00 [16.00, 22.00]	**0.011**	19.00 [16.00, 22.00]	19.00 [16.00, 22.50]	0.878
ALT (U/L)*	15.00 [12.00, 20.00]	14.00 [11.00, 18.00]	14.00 [11.00, 18.00]	0.580	14.00 [12.00, 19.00]	17.00 [13.00, 23.00]	**< 0.001**
AST/ALT*	1.24 [1.00, 1.50]	1.38 [1.16, 1.62]	1.25 [1.08, 1.53]	**< 0.001**	1.24 [1.06, 1.47]	1.09 [0.86, 1.33]	**< 0.001**
UA (μmol/L)	331.08 ± 76.24	307.38 ± 66.43	337.13 ± 76.10	**< 0.001**	327.70 ± 74.06	350.45 ± 79.38	**< 0.001**
hsCRP (mg/L)	1.98 ± 4.55	1.45 ± 3.40	1.77 ± 2.80	0.667	2.53 ± 8.21	2.37 ± 4.39	**0.039**
ALP (U/L)*	71.51 [60.15, 84.64]	69.49 [58.60, 81.74]	70.82 [60.64, 84.83]	0.136	72.85 [60.37, 82.79]	73.22 [61.55, 87.93]	0.110
Albumin (g/L)	45.04 ± 4.70	45.45 ± 4.01	45.09 ± 6.17	0.367	44.25 ± 5.16	44.90 ± 4.30	0.104
SHBG (nmol/L) *	56.10 [40.55, 75.85]	75.45 [57.23, 96.90]	56.60 [42.33, 70.50]	**< 0.001**	58.80 [44.80, 74.20]	41.70 [32.75, 55.85]	**< 0.001**
DHEAS (μg/dL) *	99.60 [70.31, 139.08]	95.49 [69.75, 131.20]	107.25 [75.54, 153.75]	**0.006**	96.53 [67.27, 136.40]	102.90 [69.40, 139.70]	0.285
Serum testosterone and its fractions							
TT (ng/dL) *	26.00 [22.00, 33.00]	26.00 [20.00, 31.00]	26.00 [22.00, 32.00]	0.289	27.00 [22.10, 35.70]	27.00 [22.10, 34.00]	0.755
FAI *	1.66 [1.12, 2.43]	1.22 [0.86, 1.69]	1.66 [1.18, 2.32]	**< 0.001**	1.62 [1.08, 2.30]	2.24 [1.56, 3.29]	**< 0.001**
cFT (ng/dL) *	0.33 [0.24, 0.45]	0.26 [0.19, 0.35]	0.33 [0.25, 0.45]	**< 0.001**	0.33 [0.24, 0.44]	0.41 [0.31, 0.54]	**< 0.001**
cFT percentage (%) *	1.26 [1.00, 1.56]	1.01 [0.83, 1.24]	1.27 [1.06, 1.53]	**< 0.001**	1.20 [1.03, 1.44]	1.52 [1.26, 1.77]	**< 0.001**
BioT (ng/dL) *	8.00 [5.80, 11.00]	6.50 [4.80, 8.50]	8.00 [6.03, 10.98]	**< 0.001**	8.20 [5.70, 10.70]	10.00 [7.25, 13.50]	**< 0.001**
BioT percentage (%) *	30.77 [24.39, 38.00]	24.91 [20.40, 30.52]	30.78 [25.39, 36.52]	**< 0.001**	29.58 [23.87, 35.67]	36.96 [30.86, 43.27]	**< 0.001**
Life style							
Physical activities (MET/day)	24.32 ± 5.96	24.72 ± 5.96	24.63 ± 6.57	0.841	24.23 ± 5.64	23.85 ± 5.75	0.403
Current smoking #	7 (0.41%)	3 (0.53%)	1 (0.35%)	0.716	1 (0.49%)	2 (0.31%)	0.707
Current drinking # @	65 (3.81%)	20 (3.51%)	12 (4.17%)	0.631	8 (3.92%)	25 (3.89%)	0.983
History of diseases and medication							
Hypertension #	472 (27.68%)	96 (16.84%)	71 (24.65%)	**0.006**	69 (33.82%)	236 (36.70%)	0.455
Diabetes #	124 (7.27%)	29 (5.09%)	23 (7.99%)	0.093	19 (9.31%)	53 (8.24%)	0.633
Dyslipidemia #	760 (44.57%)	212 (37.19%)	142 (49.31%)	**0.001**	91 (44.61%)	315 (48.99%)	0.275
Use of estrogens #	124 (7.27%)	48 (8.42%)	28 (9.72%)	0.527	14 (6.86%)	34 (5.29%)	0.388

^*^Non-normally distributed data were expressed as median [25th, 75th percentiles], and statistical significance was estimated by using Mann-Whitney *U*-tests.

^#^Categorical variables were expressed as frequency and percentage.

^@^Excessive alcohol consumption (≥ 140 g/wk for males or ≥ 70 g/wk for females) was excluded initially.

*P* values less than 0.05 are in bold.

ALP, Alkaline phosphatase; ALT, Alanine transaminase; AST, Aspartate aminotransferase; BioT, Bioavailable testosterone; BMI, Body mass index; cFT, Calculated free testosterone; CNY, Chinese Yuan; DBP, Diastolic blood pressure; DHEAS, Dehydroepiandrosterone sulphate; FAI, Free androgen index; HDL-C, High-density lipoprotein cholesterol; HOMA-IR, Homeostasis model assessment of insulin resistance; hsCRP, High-sensitivity C-reactive protein; LDL-C, Low-density lipoprotein cholesterol; MET, Metabolic equivalent of energy; NAFLD, Nonalcoholic fatty liver disease; SBP, Systolic blood pressure; SHBG, Sex hormone binding globulin; TC, Total cholesterol; TT, Total testosterone; TG, Triglycerides; UA, Uric acid; WHR, Waist-to-hip ratio.

**Figure 2:**
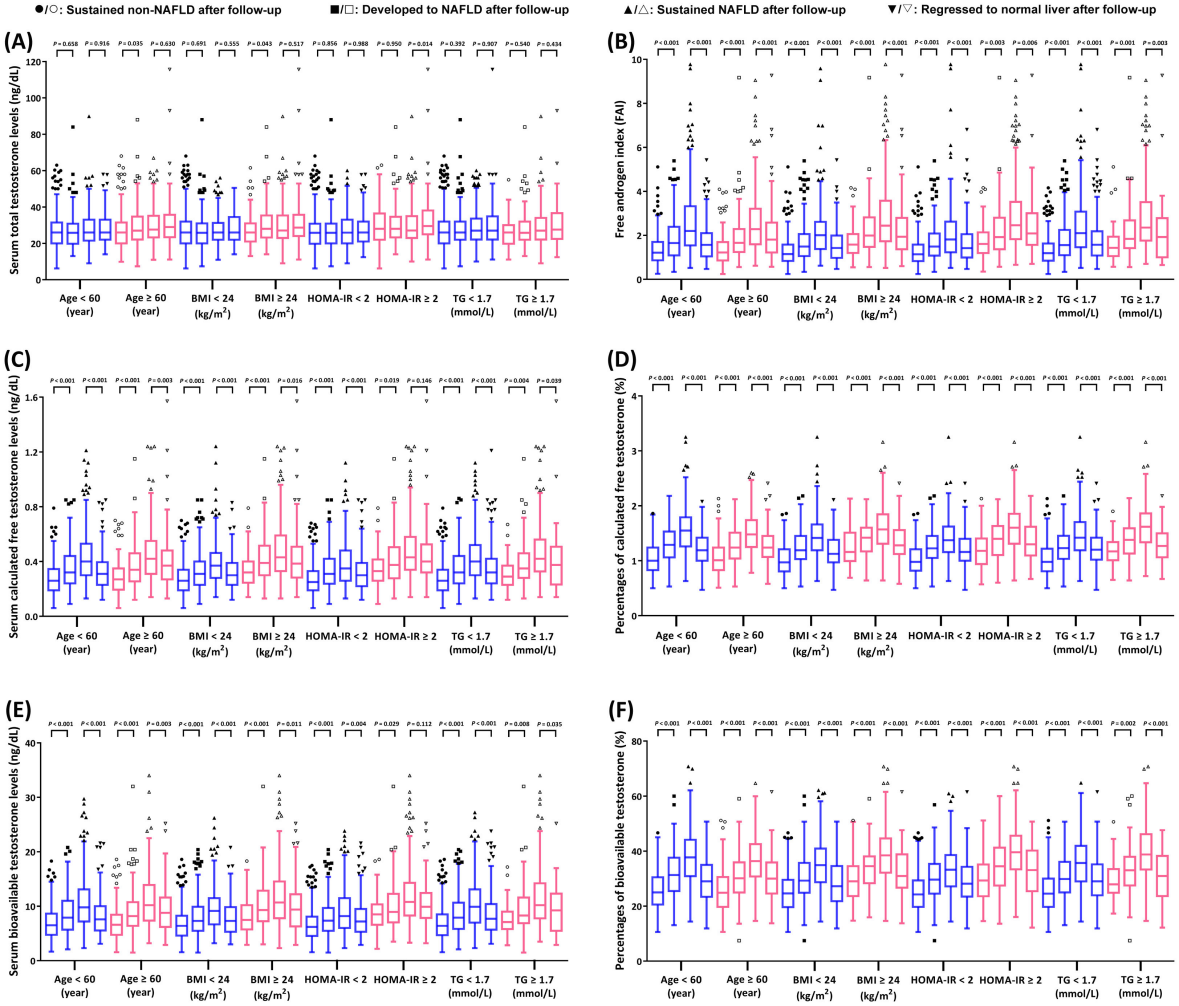
Comparison of serum testosterone levels in pre-specified subgroups according to the progression status of non-alcoholic fatty liver disease. The box plots display the median values and 25th and 75th percentiles; the whiskers represent 25th percentiles - 1.5 * interquartile range and 75th percentiles + 1.5 * interquartile range. **(A)** Serum total testosterone levels (ng/dL). **(B)** Free androgen index (FAI). **(C)** Serum calculated free testosterone levels (ng/dL). **(D)** Percentages of serum calculated free testosterone (%). **(E)** Serum bioavailable testosterone levels (ng/dL). **(F)** Percentages of serum bioavailable testosterone (%).

When comparing basal testosterone levels, postmenopausal women who developed NAFLD had higher FAI, as well as higher concentrations and percentages of both cFT and BioT (all *P-value* < 0.001). However, no significant difference in TT levels was observed (*P-value* = 0.289). As outlined in **Figure 2**, postmenopausal women who developed NAFLD had higher FAI, as well as higher concentrations and percentages of both cFT and BioT than those who sustained non-NAFLD in all subgroups (all *P-value* < 0.05).

### Clinical parameters and testosterone levels of postmenopausal women with NAFLD at baseline

On the other hand, among postmenopausal women diagnosed with NAFLD at baseline, those who regressed to normal liver after follow-up had significantly lower baseline levels of BMI, WHR, trunk fat mass, diastolic blood pressure (DBP), fasting glucose and insulin, HOMA-IR, TG, LDL-C/HDL-C ratio, ALT and UA (all *P-value* < 0.05), but had higher HDL-C, SHBG, and AST/ALT ratio (all *P-value* < 0.001) than those who sustained NAFLD after follow-up (**Table 1**).

In addition, postmenopausal women who regressed from NAFLD to normal liver had lower FAI, as well as lower concentrations and percentages of both cFT and BioT (all *P-value* < 0.001). Similarly, no significant difference in TT levels was observed (*P-value* = 0.755). When analyzing results based on subgroups, postmenopausal women who regressed to normal liver had notably lower FAI, as well as percentages and serum concentrations of both cFT and BioT, than those who sustained NAFLD (all *P-value* < 0.05) (**Figure 2**).

### Incidence and regression rates of NAFLD in postmenopausal women

**Figure 3:**
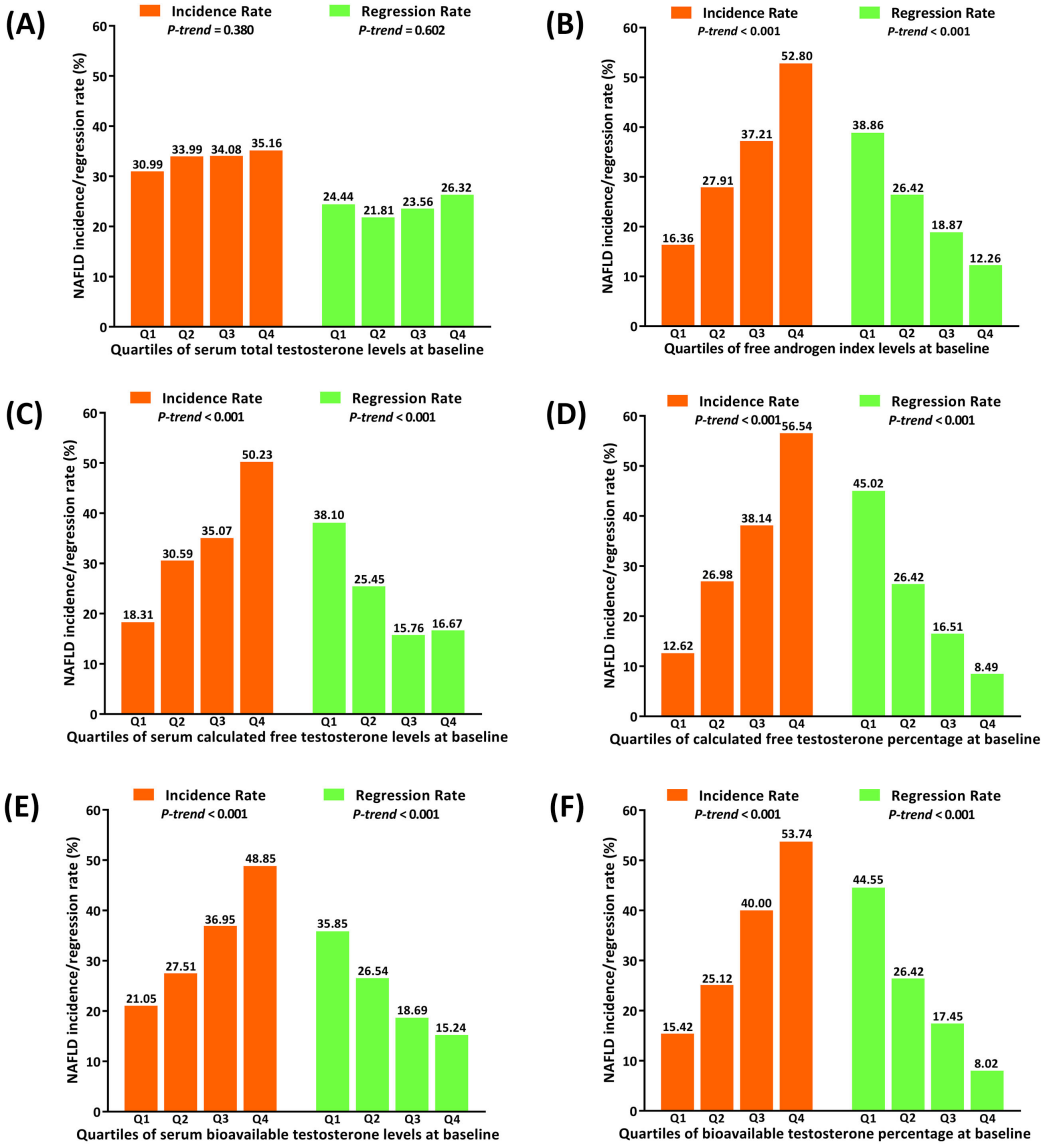
Incidence or regression rate (%) of non-alcoholic fatty liver disease by quartiles of serum testosterone levels of postmenopausal women at baseline. Data are presented as incidence rates (%) and regression rates (%) according to quartiles of baseline testosterone-related parameters. Quartile ranges for each parameter (including serum concentrations of TT, cFT, BioT, as well as %cFT, %BioT, and FAI) are provided in **Tables 2**, **3**. **(A)** Quartiles of serum total testosterone levels. **(B)** Quartiles of free androgen index (FAI). **(C)** Quartiles of serum calculated free testosterone levels. **(D)** Quartiles of serum calculated free testosterone percentages. **(E)** Quartiles of serum bioavailable testosterone levels. **(F)** Quartiles of serum bioavailable testosterone percentages. *P-value* here was analyzed using linear regression analysis.

The NAFLD incidence rate among non-NAFLD postmenopausal women over this 3-year follow-up was 33.57% (annual rate: 10.97%), while the NAFLD regression rate among postmenopausal women with NAFLD was 24.09% (annual rate: 7.90%). **Figure 3** shows the incidence rate and the regression rate of NAFLD according to the quartiles of testosterones levels. Among non-NAFLD postmenopausal women, the incidence rates increased rapidly with the incremental quartiles of FAI, as well as of concentrations and percentages of both cFT and BioT (all *P-trend* < 0.001), but not with TT levels (*P-trend* = 0.380). In contrast, among postmenopausal women with NAFLD, the regression rates decreased significantly with increasing quartiles of FAI, as well as of concentrations and percentages of both cFT and BioT (all *P-trend* < 0.001), and again, not with TT levels (*P-trend* = 0.602).

### Longitudinal analyses of the associations between baseline TT levels and NAFLD progression

First, we used multivariate logistic regression models to evaluate the associations between serum TT levels and the risk of NAFLD development or the probability of NAFLD regression. As shown in **Table 2**, there was no significant association between TT levels and NAFLD development or regression in any model (all *P-trend* > 0.05). Furthermore, when TT levels were examined as a continuous variable, the direction and magnitude of the estimates did not change appreciably.

**Table 2:** Adjusted odds ratios (95% confidence intervals) for the risk of NAFLD development and the probability of NAFLD regression after 3.05 ± 0.43 years of follow-up according to the quartiles or per 1-SD increase of serum TT levels at baseline.

Testosterone fractions	Quartiles of TT levels				*P* for trend	Per 1-SD increase
	Quartile 1	Quartile 2	Quartile 3	Quartile 4		
NAFLD development among postmenopausal women without NAFLD at baseline						
TT (ng/dL)	< 21.00 (*n* = 213)	21.00–25.99 (*n* = 203)	26.00–31.00 (*n* = 223)	> 31.00 (*n* = 219)		
Model 1 *P* values	1.000	1.146 (0.760–1.728) 0.516	1.155 (0.773–1.725) 0.483	1.205 (0.806–1.801) 0.363	0.382	1.325 (0.871–2.016) 0.189
Model 2 *P* values	1.000	1.161 (0.740–1.819) 0.516	1.065 (0.689–1.646) 0.778	1.097 (0.704–1.710) 0.683	0.795	1.224 (0.883–1.698) 0.225
Model 3 *P* values	1.000	1.132 (0.715–1.793) 0.597	1.012 (0.649–1.579) 0.957	1.045 (0.662–1.649) 0.852	0.987	1.212 (0.874–1.680) 0.250
NAFLD regression among postmenopausal women with NAFLD at baseline						
TT (ng/dL)	< 23.00 (*n* = 225)	23.00–26.99 (*n* = 188)	27.00–34.00 (*n* = 225)	> 34.00 (*n* = 209)		
Model 1 *P* values	1.000	0.875 (0.551–1.389) 0.572	0.958 (0.620–1.480) 0.846	1.091 (0.705–1.688) 0.696	0.649	0.978 (0.820–1.166) 0.801
Model 2 *P* values	1.000	0.891 (0.548–1.449) 0.642	1.041 (0.657–1.650) 0.863	1.170 (0.732–1.868) 0.512	0.444	1.038 (0.875–1.231) 0.671
Model 3 *P* values	1.000	0.890 (0.536–1.478) 0.652	1.030 (0.635–1.669) 0.906	1.193 (0.730–1.949) 0.482	0.423	1.057 (0.882–1.267) 0.549

*Univariate and multivariate logistic regression models were used to evaluate these associations both with and without adjustment for confounding variables.

Model 1: Adjusted for age and household income per month.

Model 2: Adjusted for variables in Model 1 plus body mass index, waist-to-hip ratio, physical activities (MET/day), current smoking and drinking, history of hypertension, diabetes and dyslipidemia.

Model 3: Adjusted for variables in Model 2 plus homeostasis model assessment of insulin resistance, triglycerides, total cholesterol, low-density lipoprotein cholesterol/how-density lipoprotein cholesterol ratio, alanine aminotransferase, uric acid and high-sensitivity C-reactive protein.

FAI, Free androgen index; NAFLD, Non-alcoholic fatty liver disease; SD, Standard deviation; TT, Total testosterone.

### Longitudinal analyses of the associations between baseline cFT and NAFLD progression

Next, we analyzed baseline concentrations and percentages of cFT separately, and the results are presented in **Table 3**. Among non-NAFLD postmenopausal women, the risk of developing NAFLD increased significantly with the increment in quartiles of both concentrations and percentages of cFT in all three models. The fully-adjusted ORs (95% CIs) for Q4 *vs*. Q1 in Model 3 were 2.35 (1.44–3.84, *P-trend* = 0.001) for cFT levels, and 4.58 (2.67–7.86, *P-trend* < 0.001) for %cFT. When analyzed as continuous variables, each 1-SD increase in %cFT was associated with a 55.6% increase in the risk of NAFLD development (OR = 1.56 [95% CI, 1.20–2.01], *P-value* = 0.001).

**Table 3:** Adjusted odds ratios (95% confidence intervals) for the risk of NAFLD development and the probability of NAFLD regression after 3.05 ± 0.43 years of follow-up according to the quartiles or per 1- SD increase of cFT, cFT percentage, BioT and BioT percentage at baseline.

	Quartiles of testosterone levels				*P* for trend	Per 1-SD increase
Regression models	Quartile 1	Quartile 2	Quartile 3	Quartile 4		
NAFLD development among postmenopausal women without NAFLD at baseline						
cFT (ng/dL)	< 0.21 (*n* = 213)	0.21–0.28 (*n* = 219)	0.28–0.37 (*n* = 211)	> 0.37 (*n* = 215)		
Model 1 *P* values	1.000	1.972 (1.256–3.096) 0.003	2.412 (1.541–3.776) < 0.001	4.581 (2.949–7.116) < 0.001	< 0.001	5.325 (2.975–9.532) < 0.001
Model 2 *P* values	1.000	1.608 (1.007–2.594) 0.048	1.816 (1.131–2.915) 0.014	2.758 (1.708–4.455) < 0.001	< 0.001	2.242 (1.174–4.282) 0.014
Model 3 *P* values	1.000	1.489 (0.917–2.420) 0.108	1.604 (1.007–2.602) 0.047	2.349 (1.438–3.838) 0.001	0.001	1.721 (0.913–3.244) 0.093
cFT percentage (%)	< 0.89 (*n* = 214)	0.89–1.08 (*n* = 215)	1.09–1.35 (*n* = 215)	> 1.35 (*n* = 214)		
Model 1 *P* values	1.000	2.578 (1.557–4.267) < 0.001	4.323 (2.650–7.055) < 0.001	9.324 (5.718–15.205) < 0.001	< 0.001	2.441 (1.959–3.042) < 0.001
Model 2 *P* values	1.000	2.068 (1.225–3.492) 0.007	2.697 (1.605–4.531) < 0.001	5.331 (3.176–8.949) < 0.001	< 0.001	1.723 (1.351–2.197) < 0.001
Model 3 *P* values	1.000	1.948 (1.143–3.320) 0.014	2.401 (1.411–4.086) 0.001	4.580 (2.669–7.860) < 0.001	< 0.001	1.556 (1.202–2.014) 0.001
BioT (ng/dL)	< 5.10 (*n* = 209)	5.10–6.99 (*n* = 229)	7.00–9.20 (*n* = 203)	> 9.20 (*n* = 217)		
Model 1 *P* values	1.000	1.431 (0.920–2.226) 0.112	2.198 (1.418–3.408) < 0.001	3.642 (2.374–5.587) < 0.001	< 0.001	3.544 (2.131–5.893) < 0.001
Model 2 *P* values	1.000	1.259 (0.788–2.012) 0.336	1.725 (1.081–2.751) 0.022	2.303 (1.444–3.672) < 0.001	< 0.001	1.742 (1.006–3.015) 0.047
Model 3 *P* values	1.000	1.148 (0.712–1.850) 0.571	1.493 (1.006–2.406) 0.048	1.948 (1.207–3.142) 0.006	0.003	1.417 (0.880–2.283) 0.151
BioT percentage (%)	< 21.76 (*n* = 214)	21.76–26.79 (*n* = 215)	26.80–33.03 (*n* = 215)	> 33.03 (*n* = 214)		
Model 1 *P* values	1.000	1.866 (1.151–3.025) 0.011	3.699 (2.332–5.867) < 0.001	6.591 (4.153–10.459) < 0.001	< 0.001	2.188 (1.751–2.734) < 0.001
Model 2 *P* values	1.000	1.446 (0.870–2.404) 0.155	2.502 (1.536–4.074) < 0.001	3.761 (2.294–6.166) < 0.001	< 0.001	1.545 (1.208–1.975) 0.001
Model 3 *P* values	1.000	1.357 (0.809–2.275) 0.247	2.204 (1.339–3.630) 0.002	3.134 (1.874–5.240) < 0.001	< 0.001	1.361 (1.053–1.761) 0.019
NAFLD regression among postmenopausal women with NAFLD at baseline						
cFT (ng/dL)	< 0.29 (*n* = 210)	0.29–0.39 (*n* = 224)	0.40–0.52 (*n* = 203)	> 0.52 (*n* = 210)		
Model 1 *P* values	1.000	0.551 (0.365–0.830) 0.004	0.303 (0.189–0.486) < 0.001	0.322 (0.203–0.509) < 0.001	< 0.001	0.338 (0.210–0.544) < 0.001
Model 2 *P* values	1.000	0.622 (0.403–0.959) 0.032	0.358 (0.216–0.592) < 0.001	0.441 (0.267–0.729) 0.001	< 0.001	0.471 (0.283–0.784) 0.004
Model 3 *P* values	1.000	0.702 (0.447–1.102) 0.124	0.408 (0.243–0.687) 0.001	0.558 (0.329–0.946) 0.030	0.005	0.609 (0.362–1.023) 0.061
cFT percentage (%)	< 1.19 (*n* = 211)	1.19–1.42 (*n* = 212)	1.42–1.71 (*n* = 212)	> 1.71 (*n* = 212)		
Model 1 *P* values	1.000	0.441 (0.293–0.663) < 0.001	0.243 (0.154–0.382) < 0.001	0.114 (0.066–0.200) < 0.001	< 0.001	0.398 (0.323–0.491) < 0.001
Model 2 *P* values	1.000	0.512 (0.333–0.787) 0.002	0.278 (0.173–0.447) < 0.001	0.164 (0.091–0.294) < 0.001	< 0.001	0.452 (0.362–0.565) < 0.001
Model 3 *P* values	1.000	0.592 (0.380–0.922) 0.020	0.320 (0.195–0.526) < 0.001	0.226 (0.121–0.422) < 0.001	< 0.001	0.508 (0.401–0.644) < 0.001
BioT (ng/dL)	< 7.00 (*n* = 212)	7.00–9.59 (*n* = 211)	9.60–13.00 (*n* = 214)	> 13.00 (*n* = 210)		
Model 1 *P* values	1.000	0.643 (0.424–0.974) 0.037	0.412 (0.264–0.644) < 0.001	0.319 (0.199–0.511) < 0.001	< 0.001	0.345 (0.219–0.543) < 0.001
Model 2 *P* values	1.000	0.709 (0.457–1.099) 0.124	0.481 (0.298–0.776) 0.003	0.432 (0.260–0.719) 0.001	< 0.001	0.464 (0.286–0.755) 0.002
Model 3 *P* values	1.000	0.794 (0.502–1.256) 0.325	0.555 (0.338–0.909) 0.019	0.535 (0.314–0.913) 0.022	0.008	0.578 (0.349–0.956) 0.033
BioT percentage (%)	< 28.65 (*n* = 211)	28.65–35.18 (*n* = 212)	35.19–42.14 (*n* = 212)	> 42.14 (*n* = 212)		
Model 1 *P* values	1.000	0.448 (0.297–0.674) < 0.001	0.265 (0.169–0.414) < 0.001	0.110 (0.062–0.193) < 0.001	< 0.001	0.405 (0.331–0.495) < 0.001
Model 2 *P* values	1.000	0.498 (0.325–0.763) 0.001	0.321 (0.200–0.515) < 0.001	0.156 (0.086–0.282) < 0.001	< 0.001	0.457 (0.369–0.566) < 0.001
Model 3 *P* values	1.000	0.593 (0.381–0.922) 0.020	0.364 (0.222–0.595) < 0.001	0.215 (0.115–0.403) < 0.001	< 0.001	0.503 (0.400–0.631) < 0.001

*Univariate and multivariate logistic regression models were used to evaluate these associations both with and without adjustment for confounding variables.

Model 1: Adjusted for age and household income per month.

Model 2: Adjusted for variables in Model 1 plus body mass index, waist-to-hip ratio, physical activities (MET/day), current smoking and drinking, history of hypertension, diabetes and dyslipidemia.

Model 3: Adjusted for variables in Model 2 plus homeostasis model assessment of insulin resistance, triglycerides, total cholesterol, low-density lipoprotein cholesterol/how-density lipoprotein cholesterol ratio, alanine aminotransferase, uric acid and high-sensitivity C-reactive protein.

BioT, Bioavailable testosterone; cFT, Calculated free testosterone; NAFLD, Non-alcoholic fatty liver disease; SD, Standard deviation.

On the contrary, among postmenopausal women who had NAFLD at baseline, both concentrations and percentages of cFT were negatively associated with the probability of NAFLD regression. The fully-adjusted ORs (95% CIs) for Q4 *vs*. Q1 in Model 3 were 0.56 (0.33–0.95, *P-trend* = 0.005) for cFT levels, and 0.23 (0.12–0.42, *P-trend* < 0.001) for %cFT. Furthermore, each 1-SD increase in %cFT was associated with a 49.2% decrease in the probability of NAFLD regression (OR = 0.51 [95% CI, 0.40–0.64], *P-value* < 0.001).

### Longitudinal analyses of the associations between baseline BioT and NAFLD progression

Similarly, both BioT and %BioT were positively associated with the risk of developing NAFLD among postmenopausal women without NAFLD at baseline in all three models (**Table 3**). The fully-adjusted ORs (95% CIs) for Q4 *vs*. Q1 were 1.95 (1.21–3.14, *P-trend* = 0.003) for BioT levels, and 3.13 (1.87–5.24, *P-trend* < 0.001) for %BioT. When analyzed as continuous variables, each 1-SD increase in %BioT was associated with a 36.1% increase in the risk of NAFLD development (OR = 1.36 [95% CI, 1.05–1.76], *P-value* = 0.019).

Meanwhile, among postmenopausal women with NAFLD, the probability of NAFLD regression decreased significantly with increasing quartiles of both concentrations and percentages of BioT. The fully-adjusted ORs (95% CIs) for Q4 *vs*. Q1 were 0.54 (0.31–0.91, *P-trend* = 0.008) for BioT levels, and 0.22 (0.12–0.40, *P-trend* < 0.001) for %BioT. In addition, each 1-SD increase in %BioT was associated with a 49.7% decrease in the probability of NAFLD regression (OR = 0.50 [95% CI, 0.40–0.63], *P-value* < 0.001).

### Subgroup analysis

**Figure 4:**
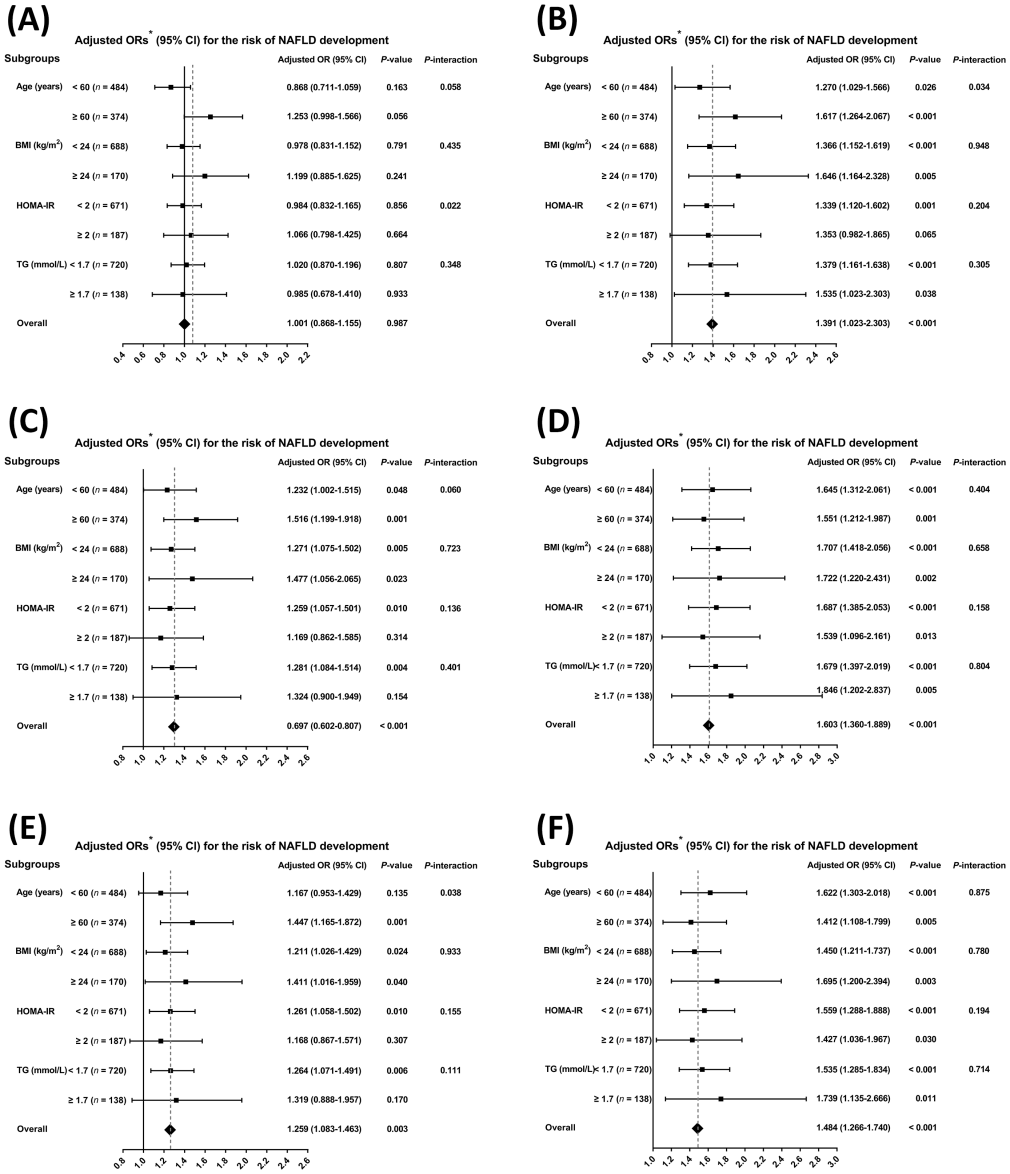
Subgroup analyses for the risk of non-alcoholic fatty liver disease development by age (< 60 *vs*. ≥ 60, years), body mass index (BMI, < 24 *vs*. ≥ 24, kg/m^2^), homeostasis model assessment of insulin resistance (HOMA-IR, < 2 *vs*. ≥ 2), and triglyceride (TG, < 1.7 *vs*. ≥ 1.7, mmol/L) using multivariable logistic regression. Data are shown as the fully-adjusted odds ratio (95% confidence interval) in Model 3 of quartiles of each testosterone component for the risk of non-alcoholic fatty liver disease development. **(A)** Total testosterone. **(B)** Free androgen index (FAI). **(C)** Calculated free testosterone. **(D)** Percentages of calculated free testosterone. **(E)** Bioavailable testosterone. **(F)** Percentages of bioavailable testosterone.

**Figure 5:**
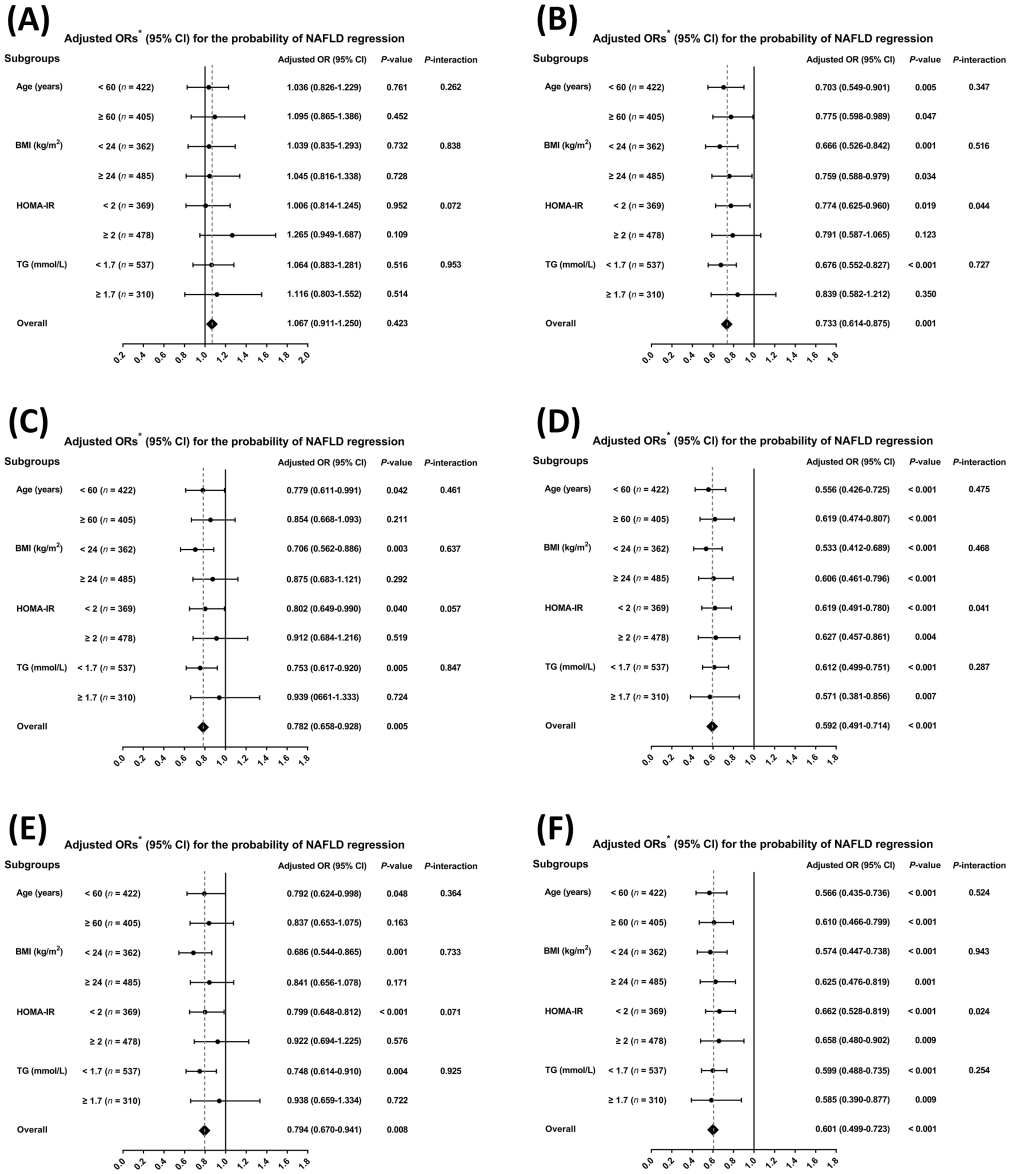
Subgroup analyses for the probability of non-alcoholic fatty liver disease regression by age (< 60 *vs*. ≥ 60, years), body mass index (BMI, < 24 *vs*. ≥ 24, kg/m^2^), homeostasis model assessment of insulin resistance (HOMA-IR, < 2 *vs*. ≥ 2), and triglyceride (TG, < 1.7 *vs*. ≥ 1.7, mmol/L) using multivariable logistic regression. Data are shown as the fully-adjusted odds ratio (95% confidence interval) in Model 3 of quartiles of each testosterone component for the probability of non-alcoholic fatty liver disease regression. **(A)** Total testosterone. **(B)** Free androgen index (FAI). **(C)** Calculated free testosterone. **(D)** Percentages of calculated free testosterone. **(E)** Bioavailable testosterone. **(F)** Percentages of bioavailable testosterone.

We evaluated whether these associations differed in pre-specified subgroups. As shown in **Figures 4A** and **5A**, there was no significant association between TT levels and the development or regression of NAFLD in any of the analyzed subgroups (all *P-value* > 0.05). Nevertheless, we found that both %cFT and %BioT were positively associated with NAFLD development, while negatively associated with NAFLD regression in all subgroups (all *P-value* < 0.05) (**Figures 4D, F**, **5D, F**). Although the positive associations between concentrations of cFT or BioT and the development of NAFLD, as well as the inverse associations with NAFLD regression, showed the same tendency in their respective subgroups, the associations in some subgroups were non-significant or only marginally significant (**Figures 4C, E**, **5C, E**).

### Determination of the independent predictors for NAFLD progression

The independent predictors for NAFLD development or regression were identified using multiple stepwise logistic regression analysis. Variables included in the original model were age, household income, BMI, trunk fat mass, physical activity (MET/day), current smoking and drinking, history of hypertension, diabetes and dyslipidemia, HOMA-IR, TG, total cholesterol, UA, hsCRP, DHEAS, LDL-C/HDL-C ratio, AST/ALT ratio, and quartiles of testosterone levels. We found that %cFT, together with trunk fat mass, HOMA-IR, and TG, was an independent predictor for both NAFLD development and regression (**Table 4**).

**Table 4:** Multiple stepwise logistic regression analyses of factors predicting the progression (development and regression) of NAFLD at follow-up.

NAFLD Status	Variables	OR (95% CI) ^#^	*P* value
NAFLD Development (*n* = 858)	Trunk fat mass	1.248 (1.152–1.352)	< 0.001
	HOMA-IR	1.249 (1.048–1.490)	0.013
	TG	1.618 (1.251–2.091)	< 0.001
	%cFT		
	Quartile 1	1.000	
	Quartile 2	1.904 (1.126–3.221)	0.016
	Quartile 3	2.304 (1.369–3.877)	0.002
	Quartile 4	4.110 (2.428–6.956)	< 0.001
NAFLD Regression (*n* = 847)	Trunk fat mass	0.879 (0.812–0.952)	0.001
	HOMA-IR	0.843 (0.733–0.969)	0.017
	TG	0.648 (0.509–0.825)	< 0.001
	%cFT		
	Quartile 1	1.000	
	Quartile 2	0.552 (0.361–0.844)	0.006
	Quartile 3	0.337 (0.210–0.540)	< 0.001
	Quartile 4	0.209 (0.116–0.376)	< 0.001

^#^Variables included in the original model were age, household income per month, BMI, trunk fat mass, physical activities (MET/day), current smoking and drinking, history of hypertension, diabetes and dyslipidemia, HOMA-IR, TG, TC, UA, high-sensitivity C-reactive protein, DHEAS, LDL-C/HDL-C ratio, AST/ALT ratio, and quartiles of TT, FAI, cFT, %cFT, BioT and %BioT.

NAFLD, Non-alcoholic fatty liver disease; OR, Odds ratio; BMI, Body mass index; HOMA-IR, Homeostasis model assessment of insulin resistance; AST/ALT ratio, Aspartate aminotransferase/alanine aminotransferase ratio; TG, Triglycerides; TC, Total cholesterol; LDL-C/HDL-C ratio, Low-density lipoprotein cholesterol/how-density lipoprotein cholesterol ratio; UA, Uric acid; DHEAS, Dehydroepiandrosterone sulphate; TT, Total testosterone; FAI, Free androgen index; cFT, Calculated free testosterone; %cFT, percentage of calculated free testosterone; BioT Bioavailable testosterone; %BioT, percentage of bioavailable testosterone.

## Discussion

Testosterone is a critical but enigmatic hormone for women, which acts directly as an androgen, in addition to being an obligatory precursor for the biosynthesis of estradiol (28). It exerts physiological effects on both reproductive and nonreproductive tissues in females, and its levels affect fertility, sex drive, red blood cell production, as well as muscle mass and fat distribution (29). Accumulating evidence indicates that testosterone plays a role in the progression of cardiovascular and metabolic diseases, and NAFLD is also considered a hepatic manifestation of metabolic syndrome (30–32). However, conflicting results have been obtained concerning the relationships between total levels of testosterone and NAFLD from previous investigations in women. In addition, it is important to note that a woman’s peak testosterone concentration is usually achieved in her third and fourth decades, followed by a steady decline in testosterone and its precursors with age (33). By menopause, circulating testosterone levels are at half their peak. The transition from pre- to postmenopause is associated with the emergence of many features of the metabolic syndrome, including increased intra-abdominal body fat, a shift toward a more atherogenic lipid profile, and increased glucose and insulin levels (34). We believe that the effects of declining testosterone in postmenopausal women are manifold and uncertain, and changes in the composition of circulating testosterone are still unclear. Therefore, it is necessary to examine the associations between circulating testosterone levels and the progression of NAFLD in postmenopausal women. In this study, based on the GNHS cohort, we conducted a community-based longitudinal study that enrolled 1,705 postmenopausal women. Our results showed that baseline concentrations and percentages of both cFT and BioT, rather than TT levels, were significantly higher in postmenopausal women who developed NAFLD after follow-up compared with those who did not progress, while they were prominently lower in postmenopausal women with NAFLD regression than in those who sustained NAFLD. Multivariate logistic regression analyses further demonstrated that higher concentrations and percentages of both cFT and BioT were associated with an increased risk of NAFLD development and a decreased probability of NAFLD regression. In contrast, there was no significant association between TT levels and NAFLD progression. Additionally, we found that %cFT was an independent predictor of both NAFLD development and regression.

Current observational studies have reached relatively consistent results in males, indicating a protective effect of higher testosterone levels, and suggesting that lower TT levels are associated with an increased risk of obesity, metabolic syndrome and NAFLD (30, 35, 36). However, results from investigations in females are inconsistent. A cross-sectional study analyzing data from participants in the US National Health and Nutrition Examination Survey indicated that low TT levels were associated with suspected NAFLD based on elevated ALT levels in postmenopausal women (19). Another study that enrolled 22 postmenopausal women with biopsy-proven NAFLD and 18 matched controls found that there was no significant difference in TT levels between NAFLD patients and the matched controls, while serum BioT and FAI, but not TT or cFT levels, were associated with NAFLD (20). The only prospective study retrieved so far, which included 1,052 premenopausal women, showed that their cFT levels were associated with NAFLD in midlife (37). When considering women with polycystic ovary syndrome (PCOS), the results are also inconsistent. A case-control study showed that cFT level and FAI were associated with NAFLD in a non-obese Asian cohort (21). Vassilatou et al. reported that PCOS patients with hepatic steatosis had higher FAI, but not TT levels, than patients without hepatic steatosis (22). However, another retrospective study found that TT levels and FAI in PCOS women were similar between patients with and without NAFLD (23). Furthermore, one meta-analysis of 7 observational studies (38), and another meta-analysis of 17 observational studies (39), both indicated that the presence of NAFLD is associated with high TT levels in women. In our opinion, there are several possible reasons for these inconsistent results, such as different study populations and designs, different diagnostic methods of NAFLD, limited sample sizes, cohorts based on specific diseases, and incomplete data on testosterone fractions (free or bioavailable levels).

As we know, testosterone circulates in the bloodstream in three different forms: weakly but mostly bound to serum albumin (~54%), and to a lesser extent, strongly bound to SHBG (~44%), while only a very small fraction of about 1% to 2% that is unbound (free testosterone, FT) (40). FT is one component of TT that is not bound to any transport protein, while BioT refers to the sum of FT and albumin-bound testosterone. An experimental study has found that FT is readily available to cells and that BioT can be more readily used by the body (41). Therefore, some researchers believe that levels of free and nonspecifically bound plasma hormone (commonly referred to as the bioavailable fraction) more reliably represent the bioactive hormone at the tissue level and generally reflect the clinical situation more accurately than total hormone levels in plasma (26, 42). This theory may explain why some patients have the same TT levels but considerably different levels of FT or BioT. However, in current routine clinical diagnosis, FT concentrations are usually evaluated only when the TT test yields doubtful results or in women with libido disorder or virilization symptoms. Nowadays, an increasing number of population-based studies have already confirmed the association between circulating FT or BioT and obesity, diabetes, and cardiometabolic diseases in females. Data from the Study of Women’s Health Across the Nation Fat Patterning Study indicated that BioT was independently associated with visceral fat (43). Olszanecka et al. found that serum cFT was associated with a worse metabolic profile, subclinical atherosclerosis and impaired diastolic function in middle-aged hypertensive women (44). Ouyang et al. reported that results from the Multi-Ethnic Study of Atherosclerosis showed that BioT, rather than TT, was associated with the extent of coronary artery calcification in postmenopausal women (45). Besides, a prospective study of 639 postmenopausal women revealed that BioT had a U-shaped association with incident coronary heart disease (46). Remarkably, the *Williams Textbook of Endocrinology* also indicates that circulating FT is a more sensitive indicator of androgen activity than total levels (40). Results from the European Male Ageing Study found that low FT, even in the presence of normal TT levels, was associated with androgen deficiency-related symptoms, while normal cFT, despite low TT levels, was not associated with these symptoms (47). However, regarding NAFLD, existing studies have mostly focused solely on TT levels when discussing associations with the disease. In this prospective study, we found that both serum free and bioavailable testosterone levels, but not total levels, were positively associated with the risk of NAFLD development and negatively associated with the probability of NAFLD regression, whether treated as categorical or continuous variables.

As a major non-reproductive target organ of sex steroid action, the liver interacts in a multifaceted and bidirectional fashion with the reproductive system (48). Researchers have found that sex steroid signaling can influence hepatic endobiotic and xenobiotic metabolism and contribute to the pathogenesis of functional and structural liver disorders (49). In turn, changes in liver function can affect the reproductive axis by regulating sex steroid metabolism and transport to tissues, for instance, via SHBG (50). Meanwhile, NAFLD, as well as MASLD, overlaps quite closely with features of the metabolic syndrome, sharing a cluster of fundamental risk factors (2, 51). Consequently, the mechanisms by which sex steroids influence the pathogenesis of NAFLD are complex and multifactorial. Existing research on the mechanism of androgens in NAFLD mainly focuses on males, while this role has been less thoroughly investigated in females. Experimental studies in some animal species have indicated that prenatal exposure of female fetuses to androgens can induce postnatal hepatic steatosis and IR (52–54). This is probably associated with subsequent down-regulation of phosphoenolpyruvate carboxykinase in the adult liver and/or hepatic up-regulation of pro-inflammatory mitogen activated protein kinase 4 (52, 55). Additionally, insulin-stimulated phosphorylation of hepatic protein kinase B was downregulated in adult livers from the aforementioned androgen-exposed model, suggesting that hepatic IR had occurred (56). On the other hand, in the postnatal androgen-exposure model, female rodents developed hepatic steatosis and IR in conjunction with reproductive phenotypes that replicated some features of PCOS when administrated dihydrotestosterone (53). In summary, existing preclinical evidence in females is consistent with a role for increased circulating testosterone in promoting hepatic steatosis, inflammation and gluconeogenesis via androgen receptor signaling, in contrast to males. Whereas similar to males, estradiol is protective against hepatic steatosis, inflammation and gluconeogenesis largely via estrogen receptor α in females (57, 58). However, whether androgen effects on the liver are direct (dose-related), or indirect (mediated by whole-body adiposity) remains unclear. Notably, after menopause, estrogen levels in women decreases rapidly, resulting in a weakening of estrogen’s protective effect. Once the balance between androgens and estrogens is broken, it may promote the occurrence and development of NAFLD. In this prospective study, we found that non-NAFLD postmenopausal women who developed NAFLD, as well as those with NAFLD who sustained NAFLD after follow-up, similarly had both higher concentrations and percentages of cFT and BioT. Additionally, both of these two groups had significantly higher fasting glucose, insulin, HOMA-IR and TG levels, which are consistent with the findings of aforementioned experimental studies.

It is necessary to point out that our study has some notable limitations that merit consideration. First, selection bias is unavoidable because participants who were recruited voluntarily from a local community may have been more health conscious. Second, our study could not completely account for other potential confounding factors associated with testosterone levels or NAFLD progression; furthermore, the effects of residual confounding resulting from measurement error in the assessment of confounding factors could also not be completely eliminated. Third, we used abdominal ultrasound as a noninvasive modality to detect the degree of hepatic steatosis. Therefore, the absence of histological confirmation of hepatic steatosis could be another weakness. Nevertheless, liver biopsy is invasive and may carry a risk of complications, while other imaging diagnostic techniques such as CT and MRI are usually expensive and time consuming. Fourth, due to the low concentration of testosterone in females, employing the chemiluminescent immunoassay, rather than using liquid chromatography-mass spectrometry (LC-MS) or gas chromatography-mass spectrometry (GC-MS), to measure serum testosterone levels presents a certain limitation. Fifth, multiple stepwise regression was used to identify independent predictors of NAFLD progression. While this method is commonly employed in exploratory analyses, it carries recognized limitations, including potential overfitting and instability in variable selection. Therefore, our findings regarding independent predictors should be interpreted as hypothesis-generating rather than confirmatory, and they warrant validation in independent cohorts. Finally, because NAFLD is a chronic disease that encompasses a wide spectrum of histopathological features, the follow-up period of the present study may be insufficient for us to track the development of advanced NAFLD and its comorbidities.

In summary, this prospective cohort study conducted in a Chinese population provides clinical evidence that increasing concentrations and percentages of free and bioavailable testosterone, rather than total levels, are positively associated with the risk of NAFLD development and inversely associated with the probability of NAFLD regression among postmenopausal women. Furthermore, both %cFT and %BioT may serve as noninvasive biomarkers for detecting the progression of NAFLD in postmenopausal women. Additionally, testosterone may represent a potential novel therapeutic target for NAFLD. In view of these limitations, future studies with larger sample size, longer follow-up period, more diverse population, more precise testosterone detection methods and with NAFLD assessed by transient elastography, magnetic resonance spectroscopy, or biopsy are warranted to further support our findings.

## Data Availability

The data analyzed in this study is subject to the following licenses/restrictions: Datasets generated during and/or analyzed during the current study are not publicly available, but are available from the corresponding author on reasonable request. Requests to access these datasets should be directed to Yuming Chen, chenyum@mail.sysu.edu.cn.
